# Dual Drug Loaded, pH-Sensitive Metal-Organic Particles for Synergistic Cancer Therapy

**DOI:** 10.3389/fbioe.2022.945148

**Published:** 2022-07-12

**Authors:** Shichao Wu, Shuo Hu, Xiangrui Yang

**Affiliations:** ^1^ Department of Nuclear Medicine, Xiangya Hospital, Central South University, Changsha, China; ^2^ Key Laboratory of Nanobiological Technology of National Health Commission, Xiangya Hospital, Central South University, Changsha, China; ^3^ National Clinical Research Center for Geriatric Disorders, Xiangya Hospital, Central South University, Changsha, China

**Keywords:** combretastatin A4 (CA4), mitoxantrone (MIT), metal-organic particles (MOPs), pH sensitive, synergistic effect

## Abstract

The strategy for dual drug-loaded nanomedicine with targeting properties was always complex. Herein, a novel strategy for the preparation of metal-organic particle-based nanomedicine has been developed, and combretastatin A4 (CA4) and mitoxantrone (MIT) loaded MOPs (CMMOPs) have been obtained. In this system, using merely Cu(II) as a bridge to connect and coordinate with the dual drugs has resulted in the CMMOPs possessing a fairly high drug load of almost 90%. Moreover, the coordination between Cu(II) and the drugs was stable at physiological pH but easily cleavable in the tumor acidic microenvironment, which would provide a good targeting property for CMMOPs. The *in vivo* experiments indicated that CMMOPs possessed a significantly enhanced antitumor efficiency with negligible side effects. The results suggest that CMMOPs could be a potential anticancer formulation for tumor-targeted drug delivery.

## Introduction

Combination chemotherapy with two or more kinds of drugs has been widely used in the clinic (Wu et al., 2017a; Liang et al., 2017). The combination candidates with non-overlapping toxicity were selected to avoid severe side effects (Wu et al., 2015; Yang et al., 2017; Shen et al., 2018). Nanoparticle drug delivery systems (NDDSs) were always employed to further enhance the synergistic effect and reduce toxicity (Wu et al., 2018; Li et al., 2020; Zheng et al., 2021). However, relatively higher toxicity than monotherapy was hardly avoided in the treatment. Hence, NDDS with long-circulation and targeting properties were designed and prepared for efficient and precise drug delivery (Zhang et al., 2018a; Song et al., 2018; Hu et al., 2020). In these NDDSs, various functional molecules were integrated to guide the particles to accumulate at the tumor site (Wang et al., 2017). Various targeting molecules, such as folate (Wu et al., 2017b; Hamdy Elshazly et al., 2019), methotrexate (Li et al., 2015), and hyaluronic acid (Misra, 2010; Choi et al., 2019), were selected to modify the NDDSs. They could bind with the specific receptors on the tumor cells to achieve targeted delivery. Molecules that responded to the tumor microenvironment were also employed to provide a targeting property (Misra, 2010; Zhang et al., 2016; Liang et al., 2019; Bao et al., 2021). For example, the urocanyl group modified pullulan nanoparticles that could rapidly release the CA4 and MTX in the tumor acidic microenvironment (Wang et al., 2013a). Wang et al. prepared a kind of ROS-responsive NDDS based on hydrophobic thioether bonds, which were easily oxidized to hydrophilic sulfones or sulfoxides (Wang et al., 2013b). The transformation would cause the disintegration of the nanoparticles and achieve the rapid release of the SN38.

Although the multifunctional NDDSs achieved great success in the laboratory, the integration of the functional molecules and dual drugs into the NDDSs always necessitates a complex procedure, which was difficult for industrial manufacture (Allen and Cullis, 2013). In order to address the problem, multifunctional MOPs based on the coordination between metal ions and antitumor candidates were developed for targeted cancer therapy (Jiang et al., 2017; Chu et al., 2019a). In the MOPs, the coordination served as the bridge and connection, which was stable at physiological pH but sensitive to the tumor's acidic environment (Chu et al., 2019b; Zhong and Sun, 2020). The coordination would be easily cleavable at pH 5.0, causing the disassembly of the MOPs and rapid drug release. Recently, various nanoscale MOPs designed for biomedicine applications have shown great potential for drug delivery of cancer therapy (Hao and Yan, 2017; Alsaiari et al., 2021). Zhang et al. (2018b) explored glucose oxidase and tirapazamine in MOPs for efficient colon cancer therapy. With the assistance of the MOPs, glucose oxidase could be efficiently delivered to tumor cells and exhaust endogenous glucose and O_2_, which would activate tirapazamine and produce a synergistic effect. In another study, AQ4N and DOX were integrated into MOPs for programmable pH/NIR/hypoxia-activated cancer photo-chemotherapy and largely enhanced the therapeutic efficiency (Zhang et al., 2018c). Promisingly, the MOPs composed of chemotherapeutic drugs may serve as a potential synergistic formulation for targeted cancer therapy.

**FIGURE 1:**
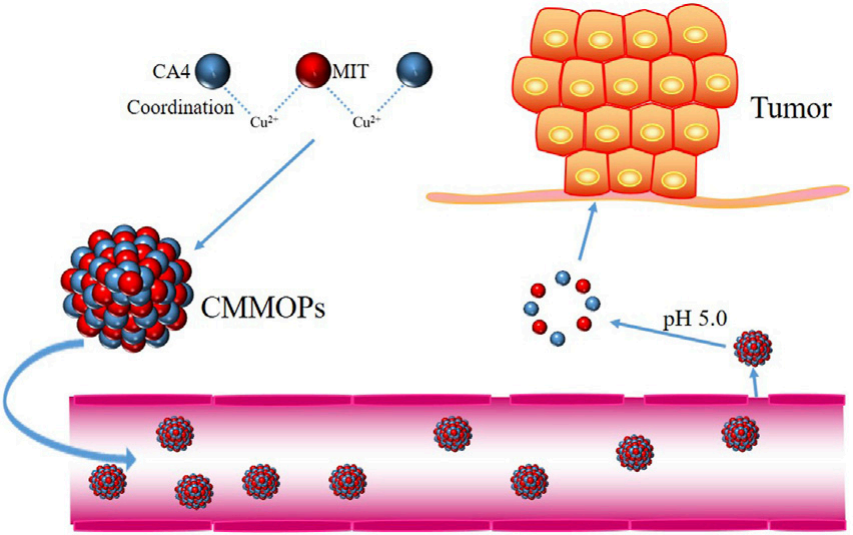
Schematic illustration of the assembly of CMMOPs and their drug delivery *in vivo*.

This study obtained CA4 and MIT-loaded MOPs *via* a one-pot process. Without complex chemical modification, CMMOPs were prepared by coordinating CA4, MIT, and Cu(II). Without a carrier, the combined drug loading of the CMMOPs was as high as 89.0%. As the coordination was easily cleavable in the tumor acidic environment, CMMOPs rapidly released CA4 and MIT at pH 5.0. The pH sensitivity provided good targeting property for CMMOPs to accumulate at the tumor site (Figure 1). Moreover, the *in vivo* experiments indicated that CMMOPs possessed a significantly enhanced antitumor efficiency with negligible side effects. The results suggest that the CMMOPs could be a potential anticancer formulation for tumor-targeted drug delivery.

## Materials and Methods

### Materials

CA4 (purity > 98%) and MIT (purity > 98%) were purchased from Shanghai Aladdin Biochemical Co., Ltd. CuCl_2_∙2H_2_O and triethylamine (TEA) were purchased from Shanghai Macklin Bio-Technology Co., Ltd. All chemicals were of analytical grade and used as received without further purification. Ultrapure water (18.2 MΩ/cm) was used throughout the work.

### Preparation and Characterization of CMMOPs

A precursor ethanol solution consisting of CA4, MIT, and CuCl_2_∙2H_2_O with a particular molar ratio (1:1:2.5) was prepared. Then, TEA was added to the precursor ethanol solution to reach pH 7.4, and the mixture was stirred for 24 h. After ultrafiltration centrifugation and washing with DI water three times, CMMOPs were finally formed. Finally, CMMOPs were redisposed in water to form CMMOPs suspension (1 mg/ml) for later use.

The morphology, energy dispersive spectroscopy (EDS) spectrum, and EDS elemental mapping of CMMOPs were examined by TEM (Tecnai G2 F20 S-TWIN) at 10 kV. The size and zeta-potential values were determined by a Malvern Zetasizer Nano-ZS machine (Malvern Instruments, Malvern). Three parallel measurements were carried out to determine the average values.

The drug loading content of CA4 and MIT in CMMOPs was determined by high-performance liquid chromatography (HPLC) at 305 and 620 nm, respectively. The drug loading content was calculated as follows:

$$\text{Drug loading content of CA}4\;\left(\text{\%}\right)=\frac{\text{weight of CA}4\text{ in CMMOPs}}{\text{weight of CMMOPs}}\times 100\text{\%},$$ (1)

$$\text{Drug loading content of MIT }\left(\text{\%}\right)=\frac{\text{weight of MIT in CMMOPs}}{\text{weight of CMMOPs}}\times 100\text{\%}.$$ (2)

### The *In Vitro* Drug Release Study of CMMOPs

The CMMOP suspension (0.1 mg/ml) was placed in a dialysis bag (MWCO 3500 Da). The dialysis bag was then immersed in PBS (150 ml, 0.15 M) at pH 5.0, 5.5, 6.0, 6.5, or 7.4 and oscillated continuously in a shaker incubator (160 rpm) at 37°C. Then, 1.0 ml of the release medium was withdrawn at predetermined time intervals and replaced with 1.0 ml of fresh release medium. All samples were assayed by HPLC.

### Animals

BALB/C mice (5–6 weeks, 18–22 g) were purchased from Hunan STA Laboratory Animal Co., Ltd. The tumor models were set up by injecting H22 cells subcutaneously in the selected position of the mice.

### Tumor Inhibition *In Vivo*

When the tumor volume of the H22 tumor-bearing mice was approximately 50 mm^3^, mice were randomly divided into five groups (12 mice per group) and treated by intravenous injection of 0.9% NaCl, the free CA4, the free MIT, the mixture of CA4 and MIT, and the CMMOPs [(CA4) = 7.3 mg/kg, (MIT) = 10.0 mg/kg] every 3 days three times. The survival rate was recorded every day. The tumor volume and body weight were monitored every 3 days. The tumor volume was calculated by the following formula: tumor volume = 0.5 × length × width^2^. The highest and lowest data were discarded at last.

## Results and Discussions

### Preparation and Characterization of CMMOPs

**FIGURE 2:**
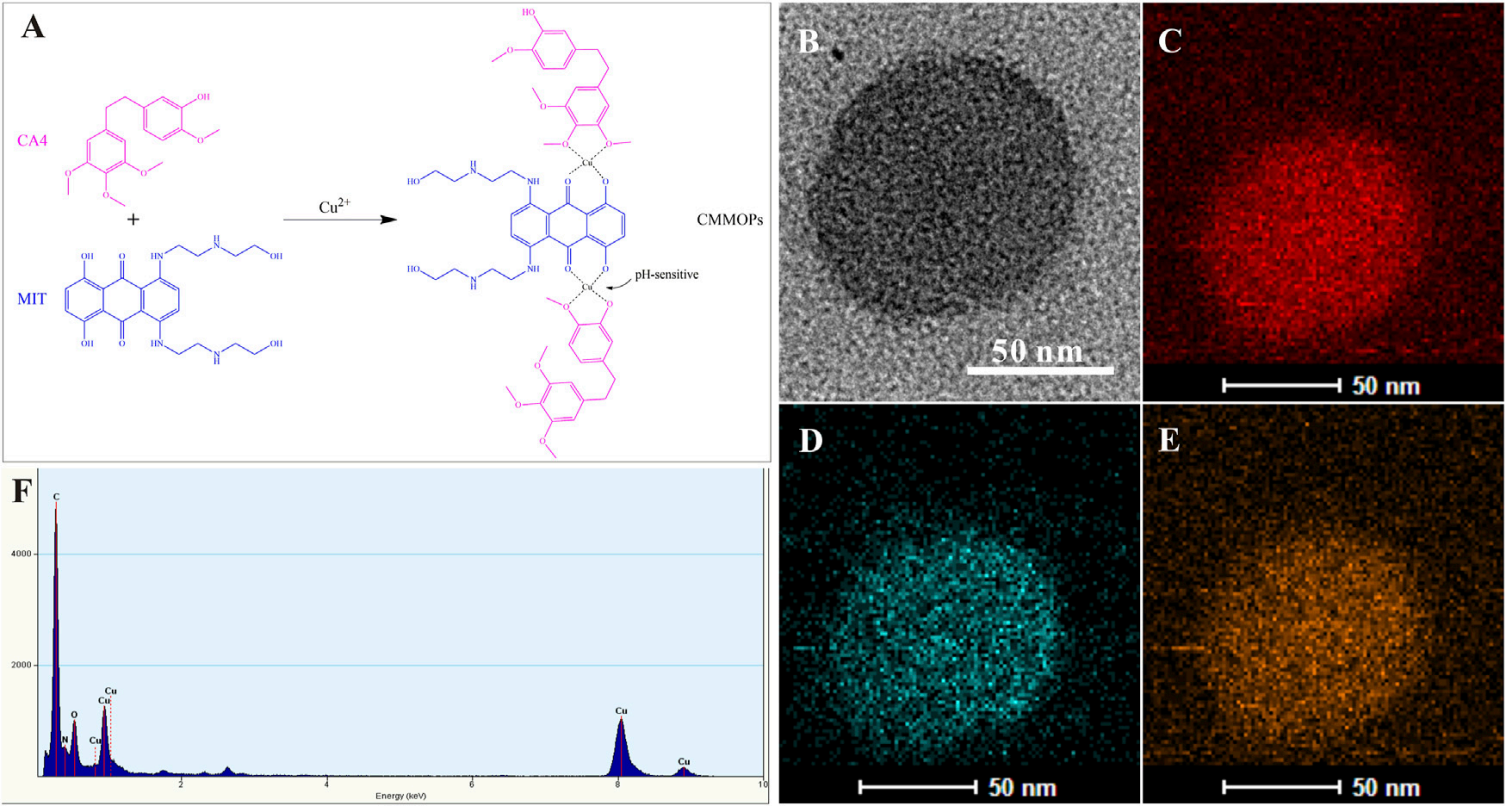
**(A)** The schematic illustration of the coordination between Cu(II) and the drugs. The TEM image **(B)**, EDS elemental mapping **(C–E)**, and the EDS spectrum **(F)** of CMMOPs.

CMMOPs were simply assembled from the hydrophobic CA4 and MIT by coordination between C-O-Cu and C=O-Cu bonds (Figure 2A). Because both drugs possessed large amounts of hydroxyl groups, they could easily coordinate to Cu(II) and form the [-CA4-Cu(II)-MIT-Cu(II)-]_n_ complex, which was dispersed in water to form CMMOPs (1 mg/ml). Then, the morphology of CMMOPs was characterized by transmission electron microscopy. The TEM image showed that CMMOPs were spherical and possessed a size of about 90 nm (Figure 2B). The energy dispersive spectroscopy (EDS) spectrum (Figure 2F) of the CMMOPs indicated the existence of Cu, C, O, and N elements in the nanoparticles. Moreover, the EDS elemental mapping (Figures 2C–E) of CMMOPs indicated the uniform distribution of Cu, C, and N elements in the nanoparticles. Results suggested that CMMOPs were assembled from the coordination of CA4, MIT, and Cu(II).

**FIGURE 3:**
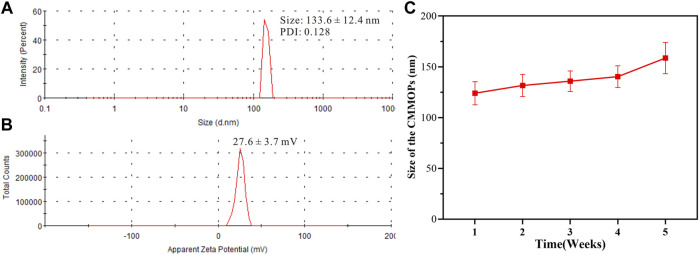
The size **(A)**, zeta potential **(B)**, and size changes **(C)** of CMMOPs.

Then, the size distribution and zeta potential of CMMOPs were obtained *via* Dynamic Light Scattering analysis. As shown in Figure 3A, CMMOPs possessed a hydrodynamic size of 133.6 ± 12.4 nm. With a zeta potential of 27.6 ± 3.7 mV (Figure 3B), the size of CMMOPs could be kept within an acceptable range over 4 weeks at 4 °C, suggesting their good stability as a nanodrug (Figure 3C). The drug loading of CA4 and MIT in the CMMOPs was 37.6% and 51.4%, respectively. Data indicated that 89.0% of CMMOPs were composed of chemotherapeutic agents, much higher than the NDDS prepared *via* traditional strategies.

### The *In Vitro* Drug Release Study of CMMOPs

**FIGURE 4:**
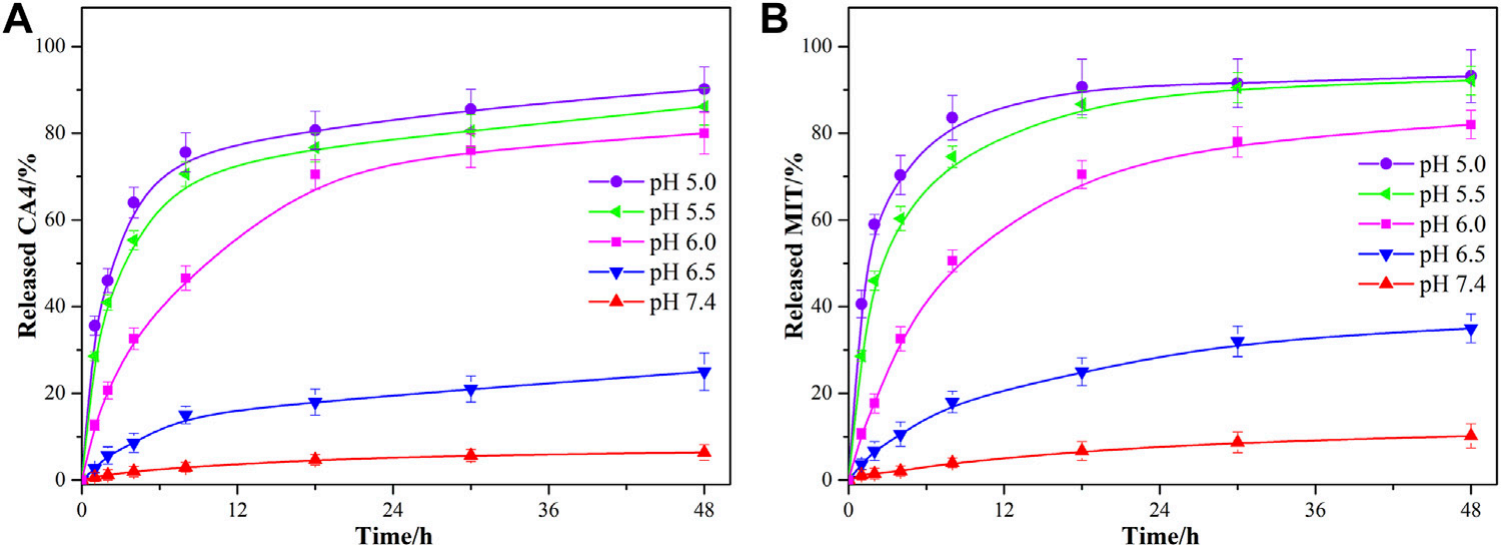
The CA4 **(A)** and MIT **(B)** release profiles of CMMOPs at pH 5.0, 5.5, 6.0, 6.5, and 7.4.

As the coordination bonds between Cu(II) and the drugs were sensitive to acid conditions, the drug release profiles of CA4 and MIT from CMMOPs in PBS under different pH conditions (pH 5.0, 5.5, 6.0, 6.5, and 7.4) were investigated. The amounts of released CA4 and MIT were determined by HPLC at pre-decided time points. As shown in Figures 4A,B, only 6.4% of the CA4 and 10.2% of the MIT were released from the CMMOPs after 48 h (pH 7.4, 37°C), indicating that CMMOPs were very stable at the pH value of the physiological condition. When reducing the pH value, both drugs were released more quickly. More strikingly, the cumulative released percentages of CA4 and MIT increased up to 90.2% and 93.2% at pH 5.0, and the burst release of the dual drugs was very sharp. Results indicated that CMMOPs would possess a slight drug release in the blood circulation but a rapid release at the tumor site. This feature could serve efficient targeting property for CMMOPs.

### Tumor Inhibition *In Vivo*

**FIGURE 5:**
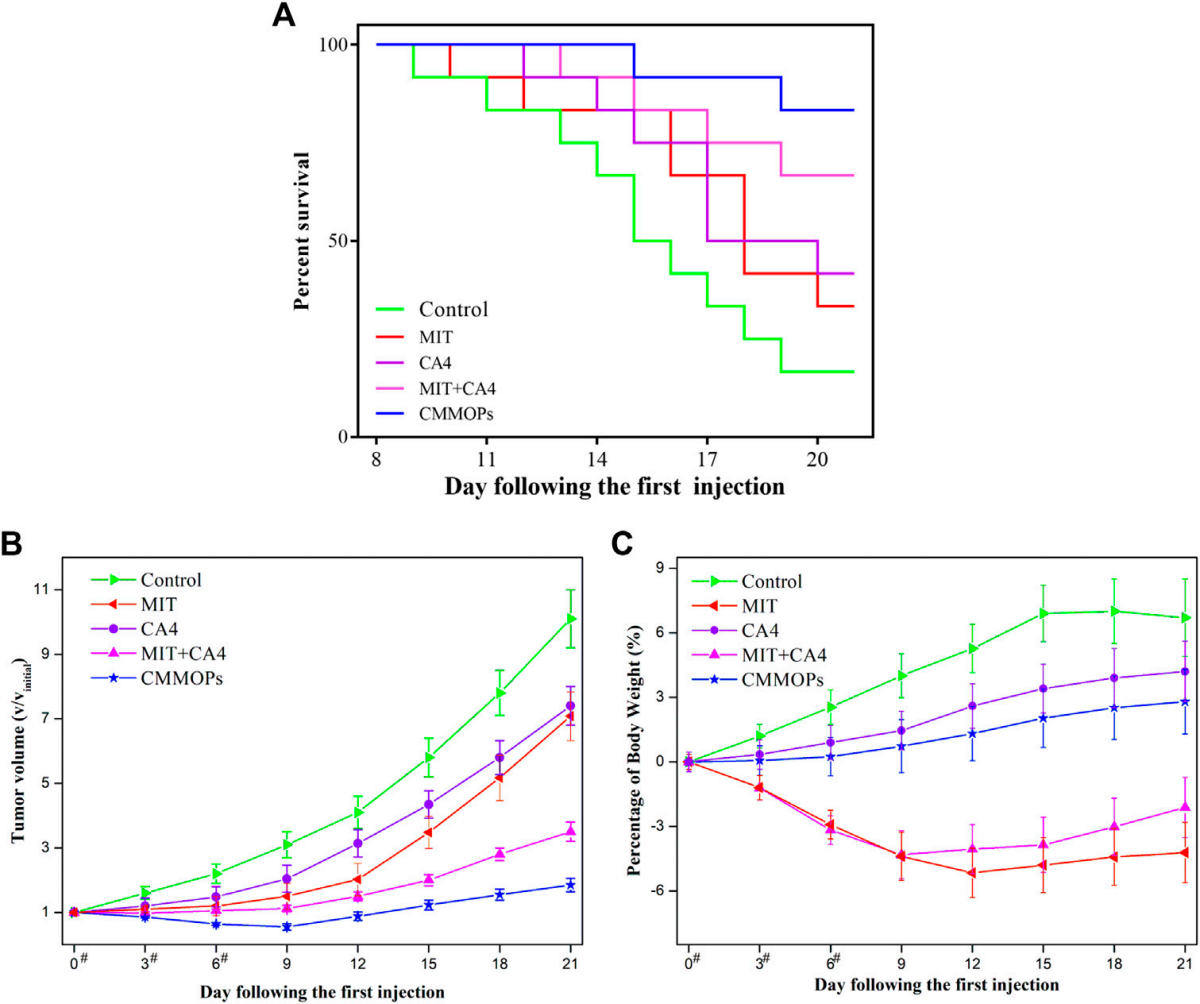
The survival rate **(A)**, tumor volume **(B)**, and body weight change **(C)** of the tumor-bearing mice injected with different formulations during the treatment (“#” represents the day on which the intravenous administration was performed).

Moreover, the *in vivo* anticancer effects of the CMMOPs were investigated by evaluating their tumor inhibition efficacy after injection. The individual drug and the physical mixture of the dual drugs were used as control. As shown in Figure 5A, only 16.7% of mice in the control group could survive for 21 days after the first drug injection. The individual administration of CA4 or MIT could improve the survival rate to 41.7% or 33.3%, indicating a certain anticancer effect. The combination of the dual drugs could largely increase the survival rate to 66.7%, suggesting the synergistic effect of the dual drugs. MIT is a cell cycle nonspecific agent, which could act directly on the tumor cells and kill them. However, CA4 is an anti-angiogenic drug that suppresses tumor growth by inhibiting angiogenesis. Dual drugs could synergistically kill the cancer cells through different routes. Furthermore, CMMOPs possessed the most pronounced inhibition effect. The survival rate of the mice injected with CMMOPs was up to 83.3%, and only two mice died from tumors in this group. The tumor volume was also recorded (Figure 5B). Tumors in the mice of the CA4 group were significantly smaller than those of the MIT group. The mice administrated with CA4 showed gradually increased body weight, while those injected with MIT possessed severe body weight loss, as well as listlessness and laziness (Figure 5C). This might be owing to the targeting and slight side effects of CA4. Although tumors in the mice administrated with the physical mixture of CA4 and MIT were much smaller than those administrated with the individual drug, the side effect of MIT was not improved. In comparison, the mice of the CMMOPs group possessed the minimum tumor volume, indicating their efficient anticancer effect. The gradually increased body weight suggested their better health conditions than those administrated with MIT formulations. More strikingly, the tumors of this group decreased with the injection of the formulation, indicating its outstanding anticancer efficacy. Overall, results forcefully indicated that CMMOPs with significant antitumor and slight side effects would greatly improve the efficacy of cancer therapy.

## Conclusion

In summary, the current study presents pH-sensitive, dual–drug-loaded metal-organic nanoparticles with enhanced anticancer efficacy. CMMOPs were obtained from the coordination between CA4, MIT, and Cu (II). The EDS elemental mapping of CMMOPs stated the uniform distribution of CA4, MIT, and Cu (II) in the nanoparticles. Drug loading of CA4 and MIT in CMMOPs was 37.6% and 51.4%, respectively. CMMOPs were stable at pH 7.4 and possessed a burst drug release at pH 5.0, which would serve the NPs’ outstanding targeting property to the tumor site. In addition to the good synergistic effect of CA4 and MIT, CMMOPs possess an excellent anticancer effect *in vivo*. The current study highlights the feasibility of metal-organic nanoparticles with a targeting property for drug delivery.

## Data Availability

The raw data supporting the conclusion of this article will be made available by the authors without undue reservation.
